# Impact of a Novel Valerian Extract on Sleep Quality, Relaxation, and GABA/Serotonin Receptor Activity in a Murine Model

**DOI:** 10.3390/antiox13060657

**Published:** 2024-05-27

**Authors:** Kazim Sahin, Hasan Gencoglu, Ahmet Kayhan Korkusuz, Cemal Orhan, İsmail Ertuğ Aldatmaz, Fusun Erten, Besir Er, Abhijeet Morde, Muralidhara Padigaru, Ertugrul Kilic

**Affiliations:** 1 Department of Animal Nutrition, Faculty of Veterinary Medicine, Firat University, 23119 Elazig, Türkiye; corhan@firat.edu.tr; 2 Department of Biology, Faculty of Science, Firat University, 23119 Elazig, Türkiye; hgencoglu@firat.edu.tr (H.G.); ber@firat.edu.tr (B.E.); 3 Department of Physiology, School of Medicine, Istanbul Medipol University, 34810 Istanbul, Türkiye; akayhankorkusuz@gmail.com (A.K.K.); ertugaldatmaz@hotmail.com (İ.E.A.); 4 Department of Veterinary Science, Pertek Sakine Genc Vocational School, Munzur University, 62500 Tunceli, Türkiye; fusunerten@munzur.edu.tr; 5 Research and Development, OmniActive Health Technologies, Mumbai 400013, India; a.morde@omniactives.com (A.M.); m.padigaru@omniactives.com (M.P.); 6 Department of Physiology, Faculty of Medicine, Istanbul Medeniyet University, 34700 Istanbul, Türkiye; kilic44@yahoo.com

**Keywords:** caffeine, melatonin, valerian, sleep disturbance, neurotransmitters, apoptosis

## Abstract

Insomnia is a major global health issue, highlighting the need for treatments that are both effective and safe. Valerian extract, a traditional remedy for sleep problems, offers potential therapeutic options. This research examined the potential sleep-enhancing effects of VA (Valerian Pdr%2) in mice. The study evaluated sleep quality by comparing the impact of the VA extract against melatonin on brain activity, using electrocorticography (ECoG) to assess changes in brain waves. For this purpose, the study utilized two experimental models on BALB/c mice to explore the effects of caffeine-induced insomnia and pentobarbital-induced sleep. In the first model, 25 mice were assigned to five groups to test the effects of caffeine (caffeine, 7.5 mg/kg i.p) alone, caffeine with melatonin (2 mg/kg), or caffeine with different doses of valerian extract (100 or 300 mg/kg) given orally on brain activity, assessed via electrocorticography (ECoG) and further analyses on the receptor proteins and neurotransmitters. In the second model, a different set of 25 mice were divided into five groups to examine the impact of pentobarbital (42 mg/kg) alone, with melatonin, or with the valerian extract on sleep induction, observing the effects 45 min after administration. The study found that ECoG frequencies were lower in groups treated with melatonin and two doses of valerian extract (100 and 300 mg/kg), with 300 mg/kg showing the most significant effect in reducing frequencies compared to the caffeine control group, indicating enhanced sleep quality (*p* < 0.05). This was supported by increased levels of serotonin, melatonin, and dopamine and higher levels of certain brain receptors in the melatonin and valerian extract groups (*p* < 0.05). Modulatory efficacy for the apoptotic markers in the brain was also noted (*p* < 0.05). Additionally, melatonin and both doses of VA increased sleep duration and reduced sleep onset time compared to the pentobarbital control, which was particularly notable with high doses. In conclusion, the findings suggest that high doses (300 mg/kg) of valerian extract enhance both the quantity and quality of sleep through the GABAergic pathway and effectively increase sleep duration while reducing the time to fall asleep in a pentobarbital-induced sleep model in mice.

## 1. Introduction

Insomnia is a widespread problem among adults [1]. Investigations from various studies indicate that between 30% and 40% of older adults experience difficulty falling or staying asleep [2,3]. Around 40% of individuals with insomnia have taken non-prescription drugs or alcoholic drinks to aid with sleep, while about one-quarter have tried prescription treatments at least once [4]. However, there is insufficient evidence for the effectiveness of regularly used drugs for insomnia such as antihistamines, chloral hydrate, barbiturates, tryptophan, and melatonin [5]. While benzodiazepines are beneficial for insomnia, the clinical benefit is minimal (less than 1 h of additional sleep) and comparable to that of exercise therapy alone [6]. Additionally, the long-term use of benzodiazepines for sleep can lead to adverse effects such as cognitive decline and a higher likelihood of motor vehicle accidents, falls, and fractures [7].

Evidence shows that herbal extracts can affect GABA receptors and regulate GABAergic signaling, although the exact way they alleviate insomnia is unknown [8]. The flowering plant valerian (*Valeriana officinalis*), a commonly used herb for anxiety and sleep problems, contains valerenic acid (VA), which activates chloride currents in certain GABA(A) receptors [9,10]. Valerian has a long history of use in Europe as a sleep aid, and its root extract is also gaining popularity as an over-the-counter remedy for insomnia in the U.S. [11]. Valerian is a therapeutic herb utilized for enhancing sleep quality and alleviating anxiety [12]. It comprises several chemicals including oils, cyclic hydrocarbons, and amino acids [13]. Valerian and cascade mixture have demonstrated efficacy in promoting quicker sleep onset, longer sleep duration, increased deep sleep, and reduced REM sleep in rodents [14]. The effects of valerian and its components (such as valerenic acid) on GABA-A receptors are similar to those of benzodiazepines, as both function as GABA agonists, which may increase the activity of the neurotransmitter GABA, inducing a decrease in brain activity [10,14].

Some evidence has suggested that valerian may also enhance the secretion of melatonin, a hormone that assists with sleep regulation, by acting as a partial agonist of the 5-hydroxytryptamine 2A receptor [15]. Moreover, it may possess mood-stabilizing and antidepressant qualities as well as anxiolytic (anxiety-reducing) effects without exhibiting sedative or myorelaxant properties [16]. In a clinical study involving human participants, researchers found that the valerian and oxazepam groups experienced enhanced sleep quality, with valerian being deemed as effective as oxazepam. Additionally, 83% of patients receiving valerian and 73% of those receiving oxazepam reported positive effects [17].

Ionotropic ligand-gated glutamate receptors GluA1, GluN1, and GluN2 are subunits that make up the NMDA (N-methyl-D-aspartate) and AMPA (α-amino-3-hydroxy-5-methyl-4-isoxazolepropionic acid) receptors, which are involved in processes such as learning, memory, and neural development [18]. While VA was shown to increase glutamate binding [19], the modulation of the GABA and NMDA receptors by VA may contribute to valerian’s anxiolytic and sedative effects. Additionally, it has been shown that in vitro valerian treatment could downregulate the apoptotic markers’ Bcl-2/Bax ratio in the ovarian cancer cell lines A2780 and OVCAR-3 in a dose-dependent manner [20]. While earlier controversial studies did not directly address the impact of sleep deprivation on sleep quality [21,22], they laid the groundwork for understanding the potential influence of sleep deprivation on apoptotic pathways and their broader implications for overall health and sleep regulation.

This research aimed to verify the impact of a novel valerinic acid (VA, Pdr%2) extract on sleep quality and its molecular action mechanisms by utilizing models of sleep induced by pentobarbital and caffeine in mice. This formula is considered a promising therapeutic option because it is affordable and has not shown any documented adverse side effects compared to other compounds [23]. Additionally, the study evaluated the serum levels of serotonin, melatonin, dopamine, malondialdehyde (MDA), the activity of antioxidant enzymes (SOD, CAT, and GPx), and the molecular effectiveness of the GABA and NMDA receptors, alongside apoptotic indicators Bax, Bcl2, and caspase-3.

## 2. Materials and Methods

### 2.1. Animals 

Fifty male BALB/c mice, aged 8 weeks and weighing 25 ± 3 g, were accommodated in a controlled environment with a 12:12-h light–dark cycle at 21 ± 1 °C and provided mice chow and water ad libitum. All mice were equally separated into two sets of experiments (Experiment I and II). All experiments in this study were conducted in compliance with the National Institute of Health (NIH) for the animal care and use of laboratory animals and approved by the local government authorities (Istanbul Medipol University Animal, Research Ethics Committee, IMU; 02.04.2021-1646). 

### 2.2. Study Design

#### Experiment I

In this experiment, 25 male BALB/c mice were randomly assigned into five groups, with five mice per group: (1) Control: mice were administered (i.p.) with a vehicle (in saline containing 5% dimethyl sulfoxide, DMSO), (2) C: mice were treated with 7.5 mg/kg caffeine, (3) C/M: mice were given caffeine and then 2 mg/kg melatonin, (4) C/VAI: mice were given caffeine followed by 100 mg/kg valerian extract, and (5) C/VAII: mice were treated with caffeine followed by 300 mg/kg valerian extract. Caffeine, melatonin, and valerian extract were dissolved in DMSO and then diluted with normal saline. The valerian doses were selected based on the previous studies [24,25]. In order to monitor the effects of caffeine and different doses of valerian extract on brain electrical activity, ECoG recordings were performed for 105 min following caffeine injection, with ECoG spike frequency and spike amplitude analysis recorded. Blood samples were rapidly collected and were centrifuged (4000× *g*; 4 °C; 15 min) for further analyses. Brains were removed and frozen on dry ice at the end of the electrocorticographic record. All samples were stored at −80 °C for analyses of the following Western blot and enzyme-linked immunosorbent assay (ELISA) kits.

All mice underwent anesthesia with urethane (1.75 g/kg, intraperitoneally, Sigma U2500, Sigma-Aldrich, St. Louis, MO, USA). Shortly afterward, they were carefully positioned within a stereotaxic apparatus (World Precision Instruments, Berlin, Germany), and their body temperatures were maintained using a rectal temperature feedback heater (507221F, Harvard Apparatus, Cambridge, MA, USA). A longitudinal incision was made on the skin following the anterior–posterior axis. The left side of the skull was delicately excised with a dental drill, ensuring the dura mater remained intact. With the bregma serving as a reference point, Ag-AgCl spherical electrodes were inserted into the epidural space of the somatomotor cortex, positioning the positive electrode 1 mm rostral and 1.5 mm lateral, and the negative electrode 3 mm caudal and 1.5 mm lateral to the bregma. The reference electrode was secured to the right hindfoot. 

Caffeine, at a dose of 7.5 mg/kg administered through intraperitoneal injection, serves as a sleep disruption agent in mice [26]. The impact of caffeine on brain function is tracked using electrocorticography (ECoG). To initiate sleep disruption, the first dose of caffeine was injected at the 15-min mark. Subsequent injections at the 30-min mark involved DMSO, melatonin, and valerian extract (VA, Valerian Pdr%2) at concentrations of 100 mg/kg and 300 mg/kg, aiming to investigate their individual effects on brain activity under conditions of sleep disturbance. The spray-dried powder form of valerian extract was used, containing a hydro-alcoholic extract of valerian roots (OmniActive Health Technologies, Mumbai, India) at 26.23%, hydroxypropyl methylcellulose at 73.27% (Novo Excipients, Navi Mumbai, India), and colloidal silicon dioxide at 0.5% (Daksh Medicare, Mumbai, India). The composition was finalized with a 2% valerenic acid content, as determined through HPLC analysis [23].

A 150-min ECoG recording was used to analyze the amplitude, spike frequency, and power spectral density. A 150-min recording of electrocorticography (ECoG) was utilized to assess the amplitude, frequency of spikes, and power spectral density. The monitoring of brain electrical activity was conducted with a PowerLab system (16/30, AD Instruments, Castle Hill, NSW, Australia) configured to sample the signals at a rate of 1000 Hz, and utilizing a band-pass filter ranging from 0.5 to 500 Hz, employing LabChart 8.1.17 software (AD Instruments, Bella Vista, NSW, Australia) for this purpose.

### 2.3. Laboratory Analyses

#### 2.3.1. Serum Hormone Levels

Enzyme-linked immunosorbent assay (ELISA) kits were employed to measure the dopamine, serotonin, and melatonin levels. The dopamine assay kit (Cat. No. E155Mo, BT-LAB, Shanghai, China) featured a detection range of 0.05–20 ng/mL and a sensitivity of 0.024 ng/mL, with an intra-assay variability under 8% and an inter-assay variability below 10%. The serotonin kit (Cat. No. MBS1601042, MyBioSource, Inc., San Diego, CA, USA) offered a range of 0.5–150 ng/mL and a sensitivity of 0.26 ng/mL with the intra- and inter-assay variabilities below 8% and 10%, respectively. For melatonin, the kit (Cat. No. E2245Mo, BT-LAB, Shanghai, China) had a range of 3–900 ng/mL and a sensitivity of 1.65 ng/mL, maintaining similar variability thresholds. Sample preparation involved lyzing and homogenizing in PBS using glass homogenizers, maintaining a cool temperature throughout the process. These neurotransmitters were measured using a microplate reader (Elx-800, Bio-Tek Instruments Inc., Winooski, VT, USA).

#### 2.3.2. Determination of Antioxidant Enzymes and Malondialdehyde (MDA)

The concentration of malondialdehyde (MDA) in the brain was determined using high-performance liquid chromatography (HPLC), while the activities of superoxide dismutase (SOD), catalase (CAT), and glutathione peroxidase (GPx) were quantified using ELISA kits. These assays for SOD, CAT, and GPx utilized commercially available kits (BT-LABS, Shanghai, China) and were performed in accordance with the manufacturer’s protocols. The detection limits for SOD (Cat. No. E2608Mo), CAT (Cat. No. E0076Mo), and GPx (Cat. No. E2106Mo) were 0.1–35 ng/mL, 0.52–70 ng/mL, and 0.1–40 ng/mL, respectively. The intra-assay and inter-assay variability were maintained below 8–10%, respectively, for all of the measured enzymes.

For the analysis of MDA, an HPLC setup was used, featuring a Shimadzu UV–Visible SPD-10 AVP detector, a CTO-10 AS VP column, and a mobile phase composed of 30 mM KH2PO4 and methanol in an 82.5:17.5 ratio (*v*/*v*, pH 3.6), flowing at a rate of 1.2 mL/min (Shimadzu, Japan). The effluent from the column was monitored at a wavelength of 250 nm. To prepare the tissue samples for analysis, they were first rinsed with PBS, minced, and then homogenized in PBS using a glass homogenizer while kept on ice. Subsequently, the homogenate was allowed to thaw at temperatures between 2 °C and 8 °C and was centrifuged at speeds ranging from 2000 to 3000 rpm for 20 min, a process applied for both the antioxidant enzyme and MDA assays.

#### 2.3.3. Western Blotting

Following the protocols established in previous research [26], brain tissue samples were collected for analyzing GABAergic receptors GABAA R2 (Cat. No. ab307359), GABAB R1 (Cat. No. ab238130), GABAB R2 (Cat. No. ab75838), serotonergic receptor 5-HT1A (Cat. No. ab85615), ionotropic glutamate receptors GluA1 (Cat. No. ab194909), GluN2A (Cat. No. ab203197), GluN1 (Cat. No, ab193310), and apoptotic markers Bax (Cat. No. ab32503), Bcl-2 (Cat. No, ab252259), and caspase-3 (Cat. No. ab13585) by Western blotting. The β-actin (Cat. No. ab8227) antibody served as the control protein for normalization. All primary and secondary antibodies utilized in the research were purchased from Abcam (Cambridge, UK) and used at a dilution range of 0.1–1 mg/mL. Briefly, tissue samples were first homogenized, then sonicated, and finally treated with a mixture of protease and phosphatase inhibitors. The total protein content was determined using a Qubit 2.0 Fluorometer according to the company’s guidelines (Invitrogen, Life Technologies Corporation, Carlsbad, CA, USA). Each sample, containing 20 µg of proteins, underwent electrophoresis for separation and was then transferred onto a nitrocellulose membrane using the Pierce Power Blotter (Thermo Scientific, Waltham, MA, USA). These membranes were incubated in a blocking solution of 5% non-fat milk in 50 mmol Tris-buffered saline with 0.1% Tween (TBS-T) for 1 h at room temperature. Blotting experiments were conducted at least three times to ensure accuracy and avoid technical errors. Protein levels were analyzed with the Image J software (version 1.54) and expressed as percentages relative to the control group, with normalization against β-actin levels.

### 2.4. Experiment II

This experiment was conducted to explore the effects of valerian extract (VA, Valerian Pdr%2) compared with melatonin on the quality of sleep by using a mouse model where sleep was induced with pentobarbital. In this context, twenty-five male BALB/c mice were divided into five groups: (1) Control group, which received saline (0.9%) followed by DMSO (99.7%), (2) P group, treated with pentobarbital alone (42 mg/kg), (3) PM group, receiving pentobarbital combined with melatonin (2 mg/kg body weight), (4) PVAI group, treated with pentobarbital and VA (100 mg/kg body weight), and (5) PVAII group, also treated with pentobarbital and VA (300 mg/kg body weight). Forty-five minutes after the oral treatments, a dose of pentobarbital (42 mg/kg) was injected into the left side of the abdomen to induce sleep in the mice. Pentobarbital, a hypnotic agent used for treating insomnia, can trigger sleep but may also disrupt sleep architecture. Sleep duration was measured as the time elapsed between reflex loss and recovery. Sleep latency was measured as the period between pentobarbital injection and sleep onset.

### 2.5. Statistical Analysis

Statistical data were evaluated using software (GraphPad Prism 10, GraphPad Software Inc. in San Diego, CA, USA). An analysis of variance (ANOVA) was conducted to assess the group differences, followed by repeated measures of ANOVA and Tukey’s HSD test. Throughout each analysis period, *p*-values below 0.05 were considered statistically significant.

## 3. Results

### 3.1. Brain Electrical Activity

**Figure 1:**
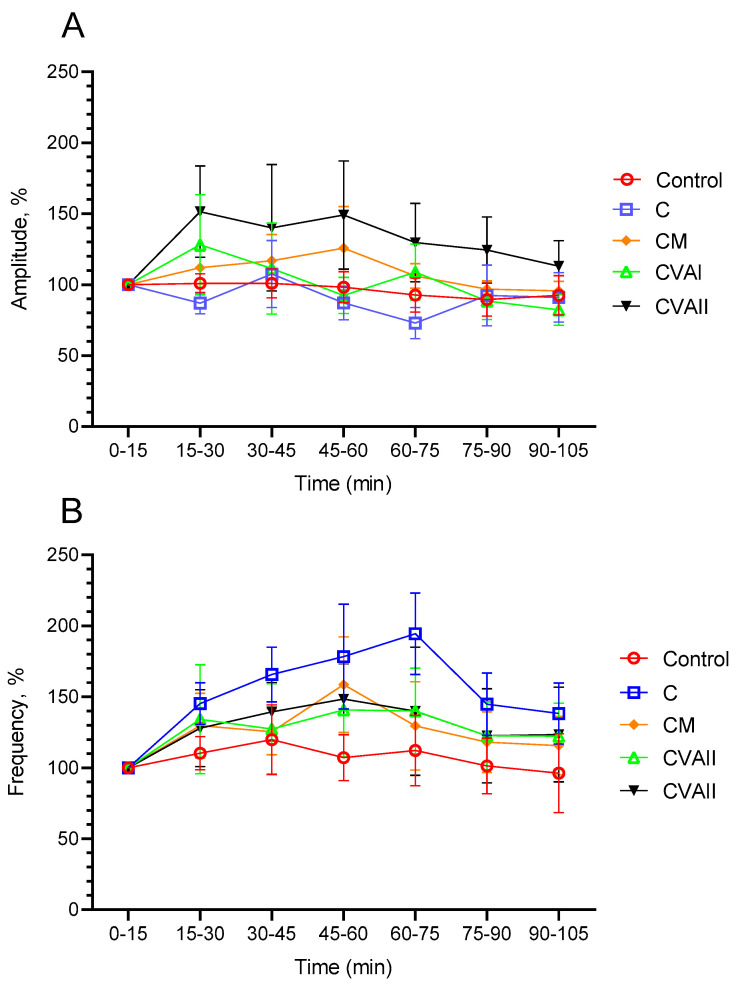
Effect of caffeine, melatonin, and VA (Valerian Pdr%2) on the brain electrical activity. (**A**,**B**) Spike frequency and amplitude analyses on the ECoG recording. Values are represented as the mean ± S.E.M. for each group, n = 5.

Figure 1 illustrates the variations in spike amplitude (A) and frequency (B) observed in the ECoG recordings, which demonstrate the effectiveness of the caffeine, melatonin, and valerian extract doses (VAI and VAII) on the brain’s electrical activity in the groups studied. In the CVAII group, the modulation of sleep states by the combined effect of caffeine and VAII was found to be higher than in the other mixtures, although it was not statistically significant (*p* > 0.05). In contrast, given caffeine’s role as a stimulant, the observation that the caffeine group (C) exhibited higher frequency values than CVAII suggests that caffeine alone prompted an increase in the rate of neural activity (*p* > 0.05). However, the reduced frequency values in CVAII imply that adding VA to caffeine might have lessened some of the caffeine’s stimulating effects, leading to a decrease in the rate of neural activity.

### 3.2. Serum Levels of Serotonin, Dopamine, and Melatonin

**Figure 2:**
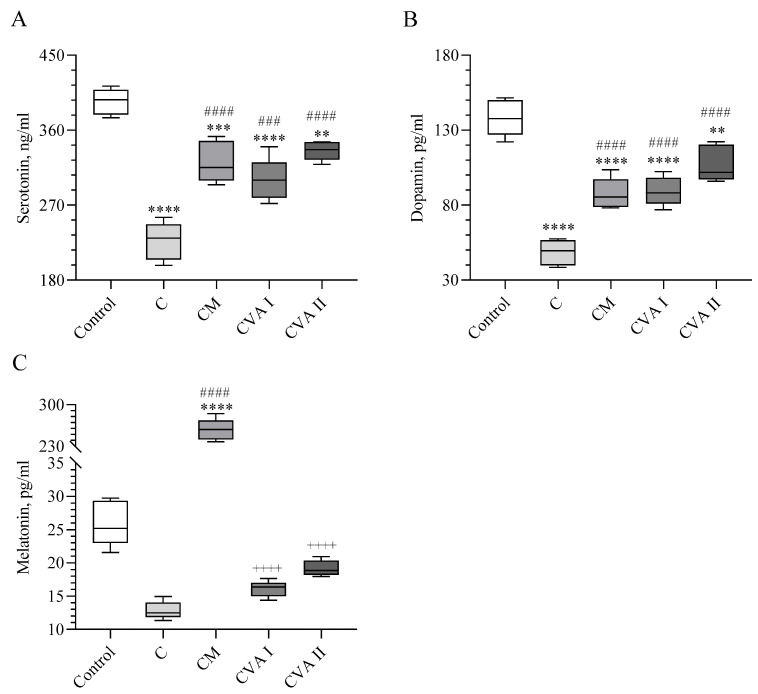
The effects of caffeine, melatonin, and VA (Valerian Pdr%2) on the serum of serotonin (**A**), dopamine (**B**), and melatonin (**C**). Data are expressed as a percent of the control value. Each bar represents the mean and standard error of the mean. Groups: Control; C: Caffeine; CM: Caffeine followed by melatonin (2 mg/kg); CVA I: Caffeine followed by VA (100 mg/kg); and CVA II: Caffeine followed by VA (300 mg/kg). ANOVA and Tukey’s post-hoc test were used for comparing the results among different treatment groups, and statistical significance between groups is shown by: ** *p* < 0.01; *** *p* < 0.001, **** *p* < 0.0001 compared to the control group, ### *p* < 0.001; #### *p* < 0.0001 compared to the C group, ++++ *p* < 0.0001 compared to the CM group.

Caffeine showed a significant decrease in the serum levels of serotonin and dopamine (*p* < 0.0001; Figure 2A,B). However, the decrease in serum melatonin caused by caffeine was not significant compared to the control (*p* > 0.05; Figure 2C). When caffeine was administered following melatonin, there was a significant increase in the serum levels of serotonin, dopamine, and melatonin (*p* < 0.0001). The highest melatonin level was noted in the CM group (*p* < 0.0001). Both CVAI and CVAII treatments led to increased serum levels of serotonin (*p* < 0.001 for CVAI, *p* < 0.0001 for CVAII) and dopamine (*p* < 0.0001 in both) when compared to the caffeine group. CVAII demonstrated a smaller decrease in the serum levels of serotonin and dopamine (*p* < 0.01), and showed no significant change in the melatonin levels compared with the control group. CVAII also recorded higher levels of serotonin, dopamine, and melatonin in serum than CVAI, with the highest serum dopamine level observed in CVAII (*p* < 0.0001), indicating that it was the most effective in mitigating the hormonal changes induced by caffeine. The lowest levels of serum serotonin and dopamine were observed in the caffeine group (*p* < 0.0001). CVAI showed the second lowest levels of serotonin and dopamine (*p* < 0.0001), 33%, respectively, suggesting levels did not significantly differ from the control but were significantly lower than those in the CM group (*p* < 0.0001).

### 3.3. Malondialdehyde (MDA) and Antioxidant Enzyme Activities

**Figure 3:**
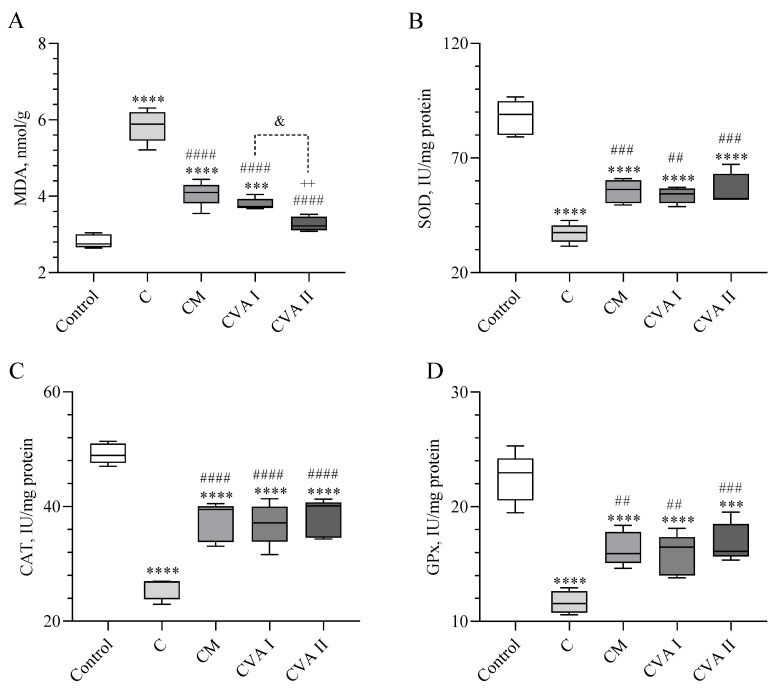
The effects of caffeine, melatonin, and VA (Valerian Pdr%2) on the brain tissue MDA (**A**), SOD (**B**), CAT (**C**), and GPx (**D**). Data are expressed as a percent of the control value. Each bar represents the mean and standard error of the mean. Groups: Control; C: Caffeine; CM: Caffeine followed by melatonin (2 mg/kg); CVA I: Caffeine followed by VA (100 mg/kg); and CVA II: Caffeine followed by VA (300 mg/kg). ANOVA and Tukey’s post-hoc test were used for comparing the results among different treatment groups, and statistical significance between groups is shown by: *** *p* < 0.001, **** *p* < 0.0001 compared to the control group, ## *p* < 0.01; ### *p* < 0.001; #### *p* < 0.0001 compared to the C group; & *p* < 0.05 compared to the CVA I group.

Figure 3 shows the concentrations of MDA in the brain, along with the activities of enzymes (SOD, CAT, and GPx). Mice that were administered caffeine alone exhibited the highest MDA levels in comparison to all of other groups (*p* < 0.0001; Figure 3A). The group that received caffeine followed by melatonin displayed reduced MDA levels relative to the caffeine group (*p* < 0.0001). Similarly, a dose of 100 mg/kg of VA had an effect on the MDA levels that was similar to that of melatonin when compared to the caffeine group as well as with a significant difference (*p* < 0.0001). Furthermore, the dose of 300 mg/kg VA resulted in a more pronounced reduction in the MDA levels than 100 mg/kg (*p* < 0.05), and was as equally effective as the dose of 100 mg/kg VA in comparison to the caffeine group and the control group (*p* < 0.0001). Figure 3B–D reveals that caffeine considerably reduced the activities of SOD, CAT, and GPx when compared to the control group. Although the groups treated with caffeine followed by melatonin, 100 mg/kg of VA, and 300 mg/kg of VA did not reach antioxidant enzyme activity levels comparable to the control for all three enzymes (*p* > 0.05), they demonstrated significantly enhanced activities of these antioxidant enzymes compared to the caffeine group (*p* < 0.01 for all). Notably, CVAII was the most effective in elevating the activities of enzymes compared to the caffeine group (*p* < 0.001), while CVAI and CM showed comparable efficacy in enhancing the CAT and GPx activities (*p* < 0.0001 and *p* < 0.01, respectively). Additionally, CVAII and CM increased the SOD activity compared to the caffeine group (*p* < 0.001), whereas CVAI presented a notable enhancement in SOD activity (*p* < 0.01).

### 3.4. GABAergic and Serotonergic Receptor Levels

**Figure 4:**
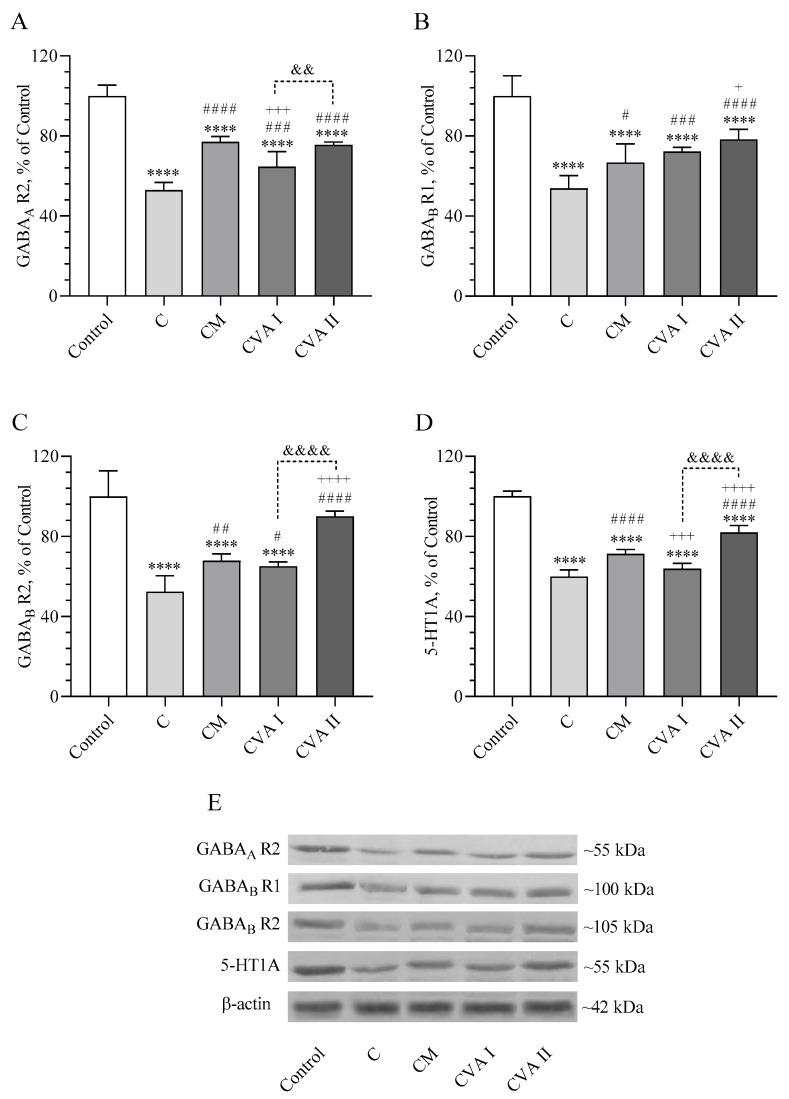
The effects of caffeine, melatonin, and VA (Valerian Pdr%2) on the brain tissue GABAergic receptors GABAA R2 (**A**), GABAB R1 (**B**), GABAB R2 (**C**), and serotonergic receptor 5-HT1A (**D**). The densitometric analysis of the relative intensity according to the control group of the Western blotting bands was performed with β-actin normalization to ensure equal protein loading (**E**). Data are expressed as a percent of the control value. Each bar represents the mean and standard error of the mean. Blots were repeated at least 3 times. Western blot analysis was performed with actin included to ensure equal protein loading. Groups: Control; C: Caffeine; CM: Caffeine followed by melatonin (2 mg/kg); CVA I: Caffeine followed by VA (100 mg/kg); and CVA II: Caffeine followed by VA (300 mg/kg). ANOVA and Tukey’s post-hoc test were used for comparing the results among different treatment groups, and statistical significance between groups is shown by: **** *p* < 0.0001 compared to control group, # *p* < 0.05; ## *p* < 0.01; ### *p* < 0.001; #### *p* < 0.0001 compared to the C group, + *p* < 0.05; +++ *p* < 0.001; ++++ *p* < 0.0001 compared to the CM group, and && *p* < 0.01; &&&& *p* < 0.0001 compared to CVA I group.

Figure 4 presents the levels of GABAergic receptors GABAAR2, GABABR1, GABABR2, and the serotonergic receptor 5-HT1A. The findings indicate that caffeine dramatically reduced the levels of these receptors in the brain compared to the control group (*p* < 0.0001). The CM (caffeine followed by melatonin) treatment resulted in a lower level of GABAAR2 compared to the control, but it was higher compared to the caffeine group (*p* < 0.0001 for both). Furthermore, the group receiving 100 mg/kg of VA (CVAI) showed increased levels of GABAAR2 compared to the caffeine group (*p* < 0.001), and the group receiving 300 mg/kg of VA (CVAII) demonstrated even higher levels than CVAI (*p* < 0.01), achieving a significant increase that was comparable to the CM group when compared to the caffeine group (*p* < 0.0001). The GABABR1 levels showed higher levels in a dose-dependent manner in CVAI and CVAII administration versus C (*p* < 0.001 and *p* < 0.0001 respectively), while also being even more effective in the CVAII group versus CM (*p* < 0.05). Similarly, the GABABR2 levels also followed this trend in improvement, with CVAI, CM, and CVAII all showing significant increases compared to the caffeine group (*p* < 0.05, *p* < 0.01, and *p* < 0.0001, respectively). Among these, CVAII demonstrated a remarkably greater enhancement in GABABR2 levels than both CM and CVAI (*p* < 0.0001). Levels of the serotonergic receptor 5-HT1A were decreased across all groups when compared to the control (*p* < 0.0001). Despite this overall decrease, the CM (caffeine followed by melatonin) and CVAII (300 mg/kg VA) groups demonstrated a significant increase in 5-HT1A levels compared to the caffeine group (C) (*p* < 0.0001). Among these, the CVAII group exhibited a notably greater increase in 5-HT1A levels compared to other groups (*p* < 0.0001), coming closest to matching the levels observed in the control group. Full immunoblots related to Figure 4 are presented as Figure S1.

### 3.5. Ionotropic Glutamate Receptor Levels

**Figure 5:**
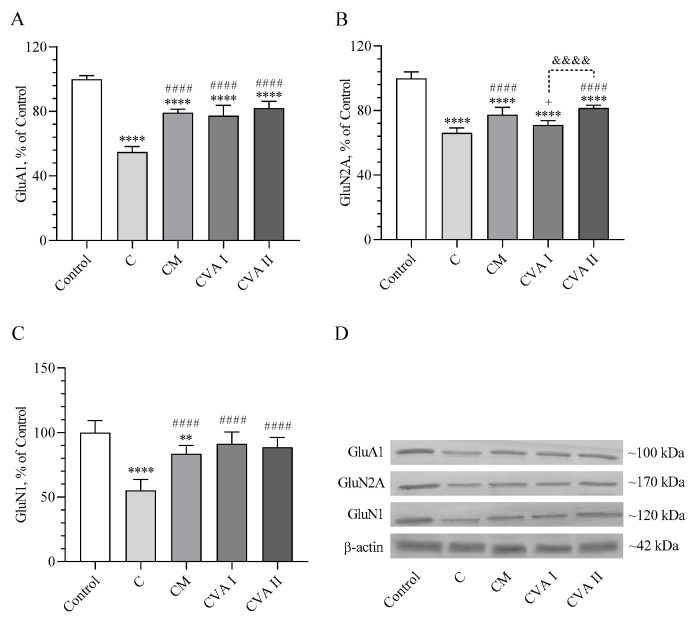
The effects of caffeine, melatonin and VA (Valerian Pdr%2) on the brain GluA1 (**A**), GluN2A (**B**), and GluN1 (**C**). The densitometric analysis of the relative intensity according to the control group of the Western blotting bands was performed with β-actin normalization to ensure equal protein loading (**D**). Data are expressed as a percent of the control value. Each bar represents the mean and standard error of the mean. Blots were repeated at least 3 times Western blot analysis was performed with actin included to ensure equal protein loading. Groups: Control; C: Caffeine; CM: Caffeine followed by melatonin (2 mg/kg); CVA I: Caffeine followed by VA (100 mg/kg); and CVA II: Caffeine followed by VA (300 mg/kg). ANOVA and Tukey’s post-hoc test were used for comparing the results among different treatment groups, and statistical significance between groups is shown by: ** *p* < 0.01; **** *p* < 0.0001 compared to the control group; #### *p* < 0.0001 compared to the C group; + *p* < 0.05 compared to the CM group; &&&& *p* < 0.0001 compared to the CVA I group.

Figure 5 illustrates the impact of various treatments on the levels of ionotropic glutamate receptors GluA1, GluN2A, and GluN1. It was found that GluA1 receptor levels decreased across all caffeine and treatment groups compared to the control (*p* < 0.0001). The decline in GluA1 levels was most significant in the caffeine group (C), whereas the application of both doses of VA and the CM (caffeine followed by melatonin) treatment resulted in an increase in GluA1 levels compared to the C group (*p* < 0.0001). While GluN2A levels also decreased in all groups compared to the control (*p* < 0.0001), the CM and CVAII groups demonstrated a significant increase in GluN2A levels compared to the caffeine group (*p* < 0.0001). Specifically, the CM group had a less marked increase in GluN2A levels compared to the CVAI group (*p* < 0.05), whereas the CVAII group showed a significant increase over the CVAI group (*p* < 0.0001). Regarding GluN1 levels, there was no significant difference between the CVAI and CVAII groups compared to the control group (*p* > 0.05). However, a decrease in GluN1 levels was observed in both the CM and caffeine groups (*p* < 0.01 and *p* < 0.0001, respectively). Adding melatonin and both doses of VA to caffeine resulted in a notable increase in GluN1 levels compared to the group that received caffeine alone (*p* < 0.0001), highlighting the potential neuroprotective effects of these treatments. Full immunoblots related to Figure 5 are presented as Figure S2

### 3.6. Apoptotic Marker Levels

**Figure 6:**
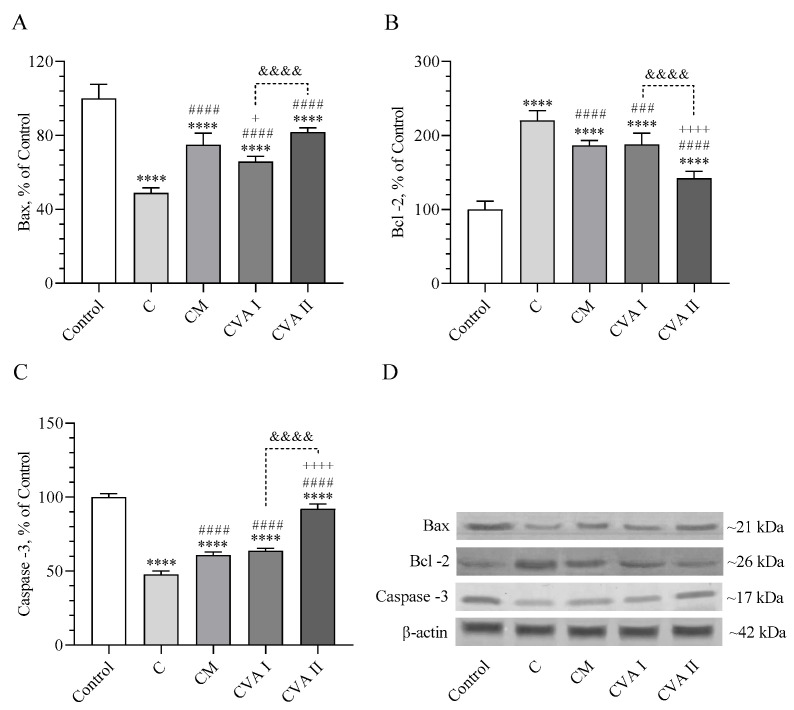
The effects of caffeine, melatonin, and VA (Valerian Pdr%2) on the brain Bax (**A**), Bcl-2 (**B**), and caspase-3 (C). The densitometric analysis of the relative intensity according to the control group of the Western blotting bands was performed with β-actin normalization to ensure equal protein loading (**D**). Data are expressed as a percent of the control value. Each bar represents the mean and standard error of the mean. Blots were repeated at least 3 times Western blot analysis was performed with actin included to ensure equal protein loading. Groups: Control; C: Caffeine; CM: Caffeine followed by melatonin (2 mg/kg); CVA I: Caffeine followed by VA (100 mg/kg); and CVA II: Caffeine followed by VA (300 mg/kg). ANOVA and Tukey’s post-hoc test were used for comparing the results among different treatment groups, and statistical significance between groups is shown by: **** *p* < 0.0001 compared to control group; ### *p* < 0.001; #### *p* < 0.0001 compared to C group; + *p* < 0.05; ++++ *p* < 0.0001 compared to CM group; &&&& *p* < 0.0001 compared to CVA I group.

Figure 6 details the effects of various treatments on apoptotic markers including Bax, Bcl-2, and caspase-3 in the brains of mice. Notably, levels of the proapoptotic protein Bax were lower in all of the treated groups compared to the control group (*p* < 0.0001), with the lowest levels observed in the caffeine group (C), indicating the highest reduction (*p* < 0.0001). However, the levels of Bax in the brains of mice treated with CM (caffeine followed by melatonin) and CVAII (300 mg/kg VA) were comparable (*p* > 0.05), with CVAII showing higher Bax levels than CVAI (100 mg/kg VA) (*p* < 0.0001). Conversely, the levels of the anti-apoptotic protein Bcl-2 were elevated in all groups compared to the control (*p* < 0.0001). The CM and CVAII groups showed a decrease in Bcl-2 levels compared to the caffeine group with a significant reduction (*p* < 0.0001), while the CVAI group also exhibited lower levels, though to a lesser extent (*p* < 0.001). The CVAII group had the lowest Bcl-2 levels, indicating a more pronounced effect than the CM and CVAI groups (*p* < 0.0001 for both). The caspase-3 levels, another apoptotic marker, were significantly lowered in all treatment groups compared to the control (*p* < 0.0001). The groups treated with CVAI, CVAII, and CM showed a significant increase in caspase-3 levels compared to the caffeine-alone group (*p* < 0.0001). There was no significant difference in caspase-3 levels between the CM and CVAI groups (*p* > 0.05), but intriguingly, the CVAII treatment demonstrated a notable increase in caspase-3 levels compared to the other treatment groups (*p* < 0.0001), suggesting distinct impacts of these treatments on apoptotic processes in the brain. Full immunoblots related to Figure 6 are presented as Figure S3.

### 3.7. Sleep Duration and Latency

**Figure 7:**
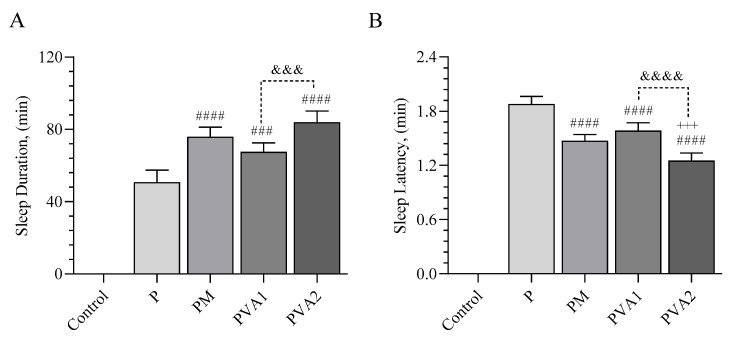
The effects of melatonin and VA (Valerian Pdr%2) on the sleep duration (**A**) and sleep latency (**B**) after pentobarbital administration. Each bar represents the mean and standard error of the mean. Groups: Control; P: Pentobarbital; PM: Pentobarbital followed by melatonin (2 mg/kg); PVA I: Pentobarbital followed by VA (100 mg/kg); and PVA II: Pentobarbital followed by VA (300 mg/kg). ANOVA and Tukey’s post-hoc test were used for comparing the results among different treatment groups, and statistical significance between groups is shown by: ### *p* < 0.001; #### *p* < 0.0001 compared to the P group; +++ *p* < 0.05 compared to the PM group; &&& *p* < 0.001; &&&& *p* < 0.0001 compared to the PVA I group.

Figure 7 demonstrates the impact of melatonin and VA (Valerian Pdr%2) on sleep duration (A) and sleep latency (B) following the administration of pentobarbital in a sleep model. The study assessed the effects of various doses of VA against the control groups including a control group, a pentobarbital (P) group, and a group receiving pentobarbital followed by melatonin (2 mg/kg) to explore their influence on sleep behaviors in mice under a pentobarbital-induced hypnotic state. Pentobarbital alone induced a sleep latency of 1.88 min. Melatonin treatment reduced this latency by 22% compared to the control, indicating a quicker sleep onset. In contrast, valerian extract treatments with CVAI and CVAII decreased sleep latency by 15% and 33%, respectively, suggesting a dose-dependent effect on accelerating sleep initiation.

Regarding sleep duration, melatonin (considered a positive control) increased it by 49% relative to the control group. Similarly, treatments with VAI and VAII significantly increased sleep duration by 33% and 65%, respectively, compared to the control. These findings highlight the beneficial effects of valerian extract on enhancing sleep quality in a pentobarbital-induced sleep model, with the 300 mg/kg dose (VAII) showing superior efficacy over the 100 mg/kg dose (VAI). Furthermore, the 100 mg/kg VA dose improved sleep duration compared to the pentobarbital group (*p* < 0.001), though it was less effective than the pentobarbital, followed by the melatonin group. Additionally, the pentobarbital-induced sleep latency was significantly reduced in the PVAI group (*p* < 0.001). The 300 mg/kg VA dose notably increased sleep duration (*p* < 0.001) and reduced sleep latency more effectively than all of other groups (*p* < 0.0001), underscoring valerian’s dose-dependent role as a relaxant that both shortens sleep latency and extends sleep duration.

## 4. Discussion

The global increase in sleep disorders has become a significant health concern, impacting not just the physical well-being of individuals, but also their mental health and overall quality of life. The intricate neurobiological mechanisms that underlie these disorders are the subject of ongoing research, which aims to shed light on the complexities of sleep regulation and to identify effective treatments for sleep-related conditions. This research is vital, considering the close association between sleep disturbances and psychiatric disorders [27]. Approximately 40% of individuals who have insomnia are also dealing with psychiatric conditions such as anxiety and depression, underscoring the interconnected nature of sleep and mental health [1]. In the quest for therapeutic options, the role of natural products is increasingly acknowledged, drawing on both traditional uses and contemporary scientific investigation [8]. *Valeriana* spp., in particular, stands out for its broad spectrum of potential therapeutic benefits. Historical usage and modern research alike highlight *Valeriana*’s efficacy not only as a sedative and sleep aid, but also for its anxiolytic, antidepressant, antispasmodic, anticancer, and anti-HIV properties [28]. Such findings are crucial for the development of holistic treatment strategies that address both the physiological and psychological aspects of sleep disorders, offering hope for safer, more natural, and potentially less side-effect-prone alternatives to conventional pharmacological treatments. The exploration of *Valeriana* spp. and similar natural compounds exemplifies the broader effort to harness the therapeutic potential of natural products in managing and treating neurological and psychiatric conditions, paving the way for advancements in health care that embrace both traditional knowledge and modern scientific insights. This research explored the complex impacts of caffeine, melatonin, and VA treatments on a range of physiological and biochemical metrics within a controlled experimental environment. It also examined how these compounds affect brain electrical activity, the serum concentrations of vital neurotransmitters, indicators of oxidative stress, receptor levels, and the sleep patterns of mice. The analysis included in this study provided valuable evaluations of these effects, offering insights into the possible therapeutic benefits of these substances.

The negative effects of caffeine on mice sleep patterns, as observed through electrocorticography (ECoG), were attributed to reduced sleep duration and increased wakefulness. However, administering melatonin and valerian counteracted the sleep disruptions caused by caffeine, with the exception of sleep patterns. Notably, all three treatments—caffeine, melatonin, and valerian—resulted in an extension of sleep duration. Moreover, the pentobarbital-induced sleep model has been recognized for its utility in evaluating sleep quality in mice, focusing on metrics such as sleep latency and duration [29]. In this study, the use of pentobarbital induced sleep disturbances, leading to shorter sleep periods and longer times to fall asleep. However, treatments with pentobarbital and melatonin restored proper sleep patterns, significantly enhancing both sleep onset and overall sleep duration in mice, echoing results from a previous study [30]. Mice treated with pentobarbital followed by 100 mg/kg of valerenic acid (VA) showed quicker sleep initiation and longer sleep compared to those only given pentobarbital, though their sleep quality did not exceed those of mice treated with pentobarbital and melatonin. Remarkably, a higher dose of VA (300 mg/kg) further improved sleep quality, as indicated by shorter sleep latency and prolonged sleep duration, outperforming the effects seen with both the melatonin and lower-dose VA treatments. This aligns with research suggesting that a blend of valerian and cascade can reduce the time to fall asleep and extend sleep duration in rodents under standard conditions and when caffeine induces arousal [14]. Overall, VA showed a dose-responsive effectiveness in enhancing sleep quality, characterized by decreased latency to sleep and extended sleep times.

Serotonin has a role in mood regulation and indirectly affects sleep by helping to produce melatonin [30]. Dopamine is involved in promoting wakefulness and alertness. Melatonin is essential for signaling the body to start and sustain sleep [31]. In line with prior research conducted on mutant rodents, pharmacogenetic results in humans provide further evidence in favor of the notion that wakefulness, sleep, and the response to stimuli are regulated reciprocally via adenosinergic and dopaminergic signaling [32]. Imbalances or disruptions in the levels or channels through which these neurotransmitters and hormones communicate can lead to sleep disorders and disturbances [33]. In the second phase of this study, the intraperitoneal injection of caffeine markedly decreased the serum levels of serotonin, dopamine, and melatonin compared to the control group. However, the co-administration of caffeine with melatonin attenuated this effect, leading to increased levels of all hormones. Conversely, both 100 and 300 mg/kg doses of VA increased the serotonin, dopamine, and melatonin levels in serum compared to the caffeine group. Similarly to these results, our earlier study showed that L-theanine could affect accordingly in terms of these hormone level changes [26].

Malondialdehyde (MDA), a product of lipid peroxidation, is widely recognized as an indicator of oxidative stress, with its elevated levels being associated with a variety of health issues such as traumatic brain injury, cancer, and cardiovascular diseases [34]. Furthermore, oxidative stress, signified by increased MDA production, can be induced by sleep deprivation, potentially leading to various detrimental health outcomes [35]. In models of caffeine-induced sleep disturbance, a significant rise in brain MDA levels suggests that caffeine may contribute to oxidative stress [26]. Nonetheless, the simultaneous administration of caffeine and melatonin has notably decreased brain MDA levels. Additionally, administering 100 and 300 mg/kg doses of VA has been shown to mitigate oxidative stress, as evidenced by elevated brain activities of SOD, CAT, and GPx. Particularly, the 300 mg/kg dose of VA resulted in lower MDA levels compared to both the caffeine-melatonin treatment and the 100 mg/kg VA treatment, though the difference was not statistically significant, suggesting that higher doses of VA might be effective in normalizing oxidative stress levels in sleep-related disorders.

Valerian extracts (VAI and VAII) and melatonin, serving as a positive control, countered the reduction in antioxidant serum levels (SOD, CAT, and GPx) in the brains of caffeine-treated mice while also reducing the MDA levels compared to mice treated with caffeine alone. However, research has shown that high doses of valerian (ranging from 500 mg to 2000 mg) can have negative effects on both somatic and germ cells in mice, potentially due to oxidative stress mechanisms, as reflected by increased MDA levels and decreased levels of nonprotein sulfhydryl groups in hepatic and testicular cells [36]. This suggests a risk of oxidative stress-induced genotoxicity and epigenetic alterations. Despite these findings, Valerian administration was not found to affect stress indicators in the liver or kidneys of rats but did restore the mRNA expression levels of SOD and CAT, showcasing its ability to counteract oxidative stress induced by substances like rotenone [37]. Additionally, a recent study found that a chitosan nano-emulsion coating infused with *Valeriana officinalis* essential oil successfully preserved the activity of enzymes such as SOD, CAT, and ascorbate peroxidase (APX), further highlighting valerian’s potential in managing oxidative stress [38].

In the present study, the significant reduction in GABAergic and serotonergic receptor levels by caffeine and their restoration by melatonin and VA treatments indicate the potential of these treatments in counteracting caffeine-induced neurochemical imbalances. VAII, in particular, showed a pronounced effect in restoring these receptor levels, highlighting its role in maintaining neural homeostasis. Specifically, the expression levels of GABAA R2 were enhanced by 22% with melatonin and 43% with VAII, GABAB R1 by 34% with melatonin and 45% with VAII, and GABAB R2 by 24% with melatonin and a remarkable 72% with VII. Additionally, the 5-HT1A receptor saw an increase of 6% with melatonin and 27% with VAII. These findings highlight the potential of melatonin and valerian in modulating neurotransmitter receptor expression in response to caffeine-induced disturbances, highlighting their therapeutic promise in neurochemical regulation. Anxiety reduction and a calming effect on the brain are considered to result from GABA binding to these receptors [39]. Valerian volatile oil was employed to treat insomnia in a manner parallel to ours; it increased the expression of 5-HT and GABA in the hippocampus of rodents by activating the serotonergic synapse signal pathway, thereby alleviating symptoms of insomnia and reducing anxiety [40]. Furthermore, the sleep-enhancing impact of the combination of valerian and cascade was demonstrated to be caused by the increased expression of the gamma-aminobutyric acid A receptor [14].

The *Valerianaceae* family has been postulated to interact with glutamatergic receptors, playing a role in regulating the sleep–wake cycle and producing anxiolytic effects [41]. Research involving two distinct valerian extracts, one aqueous and the other hydroalcoholic, analyzed their effects on rat synaptic membranes [19]. Both extracts were found to engage with glutamate receptors; however, the hydroalcoholic extract demonstrated a selective affinity for a particular receptor ligand. Over time, the stability and effectiveness of these extracts varied; initially, the aqueous extract impeded receptor binding, whereas the hydroalcoholic extract significantly facilitated it. This variability underscores the complex nature of valerian’s interaction with neural receptors, and its potential implications for sleep and anxiety regulation.

In the current study, valerian extracts VAI and VAII notably elevated the levels of glutamate receptors GluA1 (41% and 49%, respectively), GluN2A (7% and 23%, respectively), and GluN1 (65% and 61%, respectively) in the brain following caffeine administration. These findings suggest that the augmentation of glutamate receptors could contribute to enhanced sleep behavior and neurological function. This increase in glutamate receptor levels hints at the potential mechanism through which valerian extracts may exert their beneficial effects on sleep and neuroregulation in the context of caffeine-induced disturbances.

Apoptosis, also known as programmed cell death, is regulated by the proteins Bax and Bcl-2, with Bax stimulating apoptosis and Bcl-2 inhibiting it [42]. Caffeine has been shown to modulate the balance between these proteins, leading to changes in apoptotic activity [43]. Some studies suggest that caffeine may upregulate Bax expression or downregulate Bcl-2 expression, thereby promoting apoptosis in certain conditions [44]. Conversely, in other contexts, caffeine may exert neuroprotective effects by decreasing Bax expression or increasing Bcl-2 expression, thus inhibiting apoptosis and promoting cell survival [45]. The decrease in proapoptotic Bax levels and changes in Bcl-2 and caspase-3 levels across treatments underscore the complex interplay between caffeine, melatonin, and VA on the cellular survival pathways. The results suggest that these treatments can influence apoptotic processes in the brain, with VAII showing a distinct capacity to modulate these pathways, possibly offering neuroprotective benefits. Valerian has also been shown to exhibit inhibitory effects on rat hepatocarcinogenesis by inhibiting oxidative DNA damage, suppressing cell proliferation, and inducing apoptosis in GST-P+ foci by activating GABA(A)R-mediated signaling [46]. These findings suggest the protective activity of valerian and support the changes in apoptotic markers we found in the present study.

## 5. Conclusions

The study demonstrated that administering novel valerian extract doses of 100 mg/kg and 300 mg/kg to mice effectively reduced sleep latency and extended sleep duration, indicating improved sleep patterns and efficiency. This valerian extract also positively influenced neurotransmitter levels in the brain, enhancing rapid neurotransmitter regulation, nerve function, and relaxation, contributing to better sleep quality and recovery from exhaustion. Specifically, the extract increased GABA expression, a crucial inhibitory neurotransmitter for sleep regulation, and countered the sleep-disruptive effects of caffeine by blocking the adenosine receptors. Additionally, the valerian treatment upregulated important glutamate receptors (GluA1, GluN2A, and GluN1) and modulated apoptotic markers (Bax, Bcl-2, caspase-3), suggesting potential benefits for sleep behavior and neurological health. The extract also displayed antioxidant and apoptosis regulatory activities, offering neuroprotective effects by reducing oxidative stress and enhancing the clearance of neurotoxic metabolites, further underscoring its therapeutic potential for sleep improvement and CNS health. As a result, the results underscore the therapeutic potential of these natural compounds in enhancing sleep quality, modulating neurotransmitter levels, reducing oxidative stress, and possibly offering neuroprotective effects. Further research is warranted to elucidate the mechanisms underlying these effects and to explore their clinical implications.

## Data Availability

The data presented in this study are available on request from the corresponding author.

## Supplementary Materials

The following supporting information can be downloaded at: https://www.mdpi.com/article/10.3390/antiox13060657/s1, As Full immunoblots related to Figure 4, Figure 5 and Figure 6 are presented as Figures S1–S3.
