# Supplementary material for: Impact of a Novel Valerian Extract on Sleep Quality, Relaxation, and GABA/Serotonin Receptor Activity in a Murine Model
Source: Antioxidants (Basel). 2024 May 27;13(6):657. doi: 10.3390/antiox13060657 (PMC11200646; doi:10.3390/antiox13060657)
Supplement: Supplementary file 1 [file antioxidants-13-00657-s001.zip › antioxidants-2952974-supplementary.pdf]

Supplementary

Impact of an Innovative Valerian Extract on Sleep Quality, Relaxation, and GABA/Serotonin Receptor Activity in a Murine Model

Kazim Sahin [1]*, Hasan Gencoglu [2], Ahmet Kayhan Korkusuz [3], İsmail Ertuğ Aldatmaz [3], Cemal Orhan [1], Fusun Erten [4], Besir Er [2], Abhijeet Morde [5], Muralidhara Padigaru [5] and Ertugrul Kilic [6]

[1] Department of Animal Nutrition, Faculty of Veterinary Medicine, Firat University, 23119, Elazig, Turkiye; ksahin@firat.edu.tr (K.S.); corhan@firat.edu.tr (C.O.)
[2] Department of Biology, Faculty of Science, Firat University, 23119, Elazig, Turkiye; hgencoglu@firat.edu.tr (H.G.); ber@firat.edu.tr (B.E.)
[3] Department of Physiology, School of Medicine, Istanbul Medipol University, 34810, Istanbul, Turkiye; akayhankorkusuz@gmail.com (A.K.K.); ertugaldatmaz@hotmail.com (İ.E.A.)
[4] Department of Veterinary Science, Pertek Sakine Genc Vocational School, Munzur University, Tunceli, Turkiye; fusunerten@munzur.edu.tr (F.E.)
[5] Research and Development, OmniActive Health Technologies, Mumbai, India; a.morde@omniactives.com (A.A.M); m.padigaru@omniactives.com (M.P.)
[6] Department of Physiology, Faculty of Medicine, Istanbul Medeniyet University, 34700, Istanbul, Turkiye; kilic44@yahoo.com (E.K.)

* Correspondence: ksahin@firat.edu.tr; Tel.: +90-4242370000 (ext. 3938))

Citation: To be added by editorial staff during production.

Academic Editor: Firstname Lastname

Received: date
Revised: date
Accepted: date
Published: date

Copyright: © 2024 by the authors. Submitted for possible open access publication under the terms and conditions of the Creative Commons Attribution (CC BY) license (https://creativecommons.org/licenses/by/4.0/).

GABAA R2 (A)

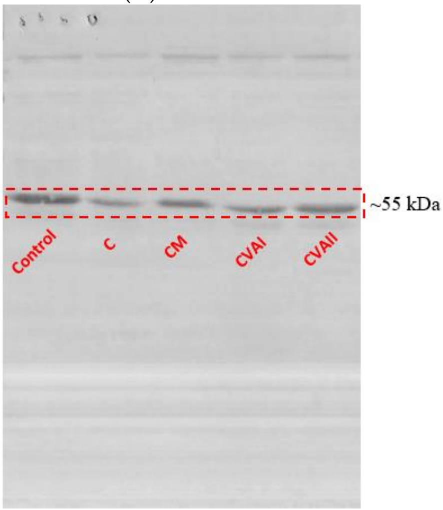

GABAB R1 (B)

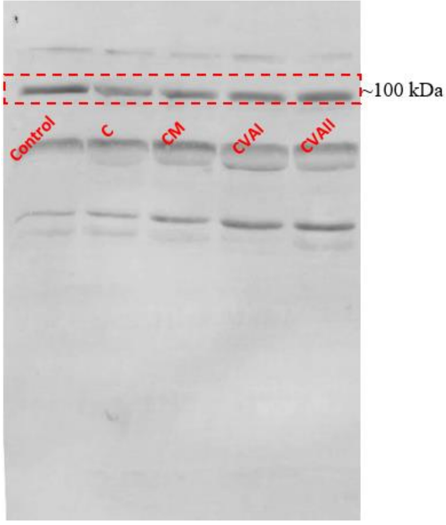

GABAB R2 (C)

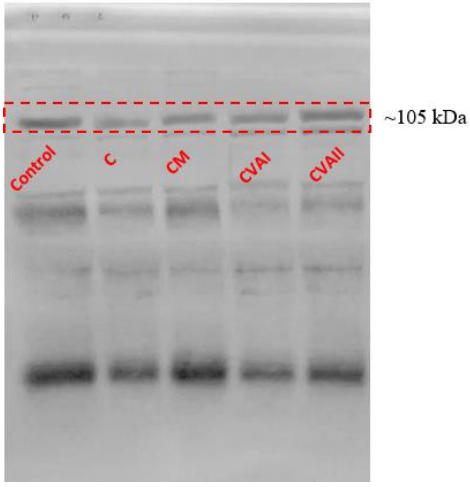

5-HT1A (D)

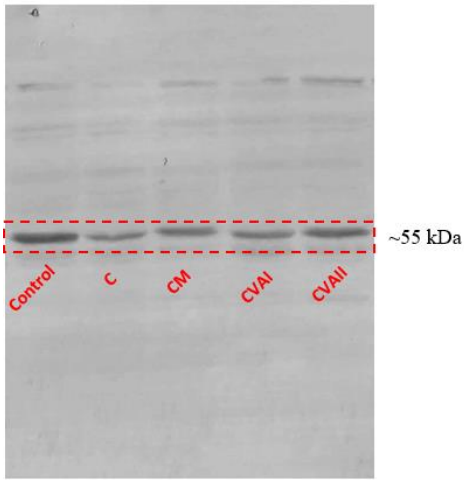

β-actin (E)

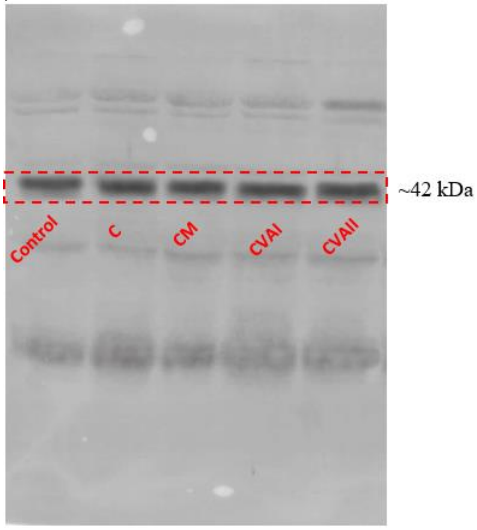

Figure S1. Full immunoblots related to Figure 4 in the main text: The effects of caffeine, melatonin, and valerian extracts (VA) on the brain tissue GABAergic receptors GABAA R2 (A), GABAB R1 (B), GABAB R2 (C), and serotonergic receptor 5-HT1A (D). The densitometric analysis of the relative intensity according to the control group of the Western blotting bands was performed with β-actin normalization to ensure equal protein loading (E). Each immunoblot is a representative of three independent experiments. Red dotted rectangles delineate the results shown in Figure 4 in the main text. M.W. (in kDa) is indicated. Groups: Control: Saline followed by saline, C: Caffeine followed by saline, CM: Caffeine followed by Melatonin (2 mg/kg), CVA I: Caffeine followed by VA-Omni (100 mg/kg), and CVA II: Caffeine followed by VA-Omni (300 mg/kg).

GluA1(A)

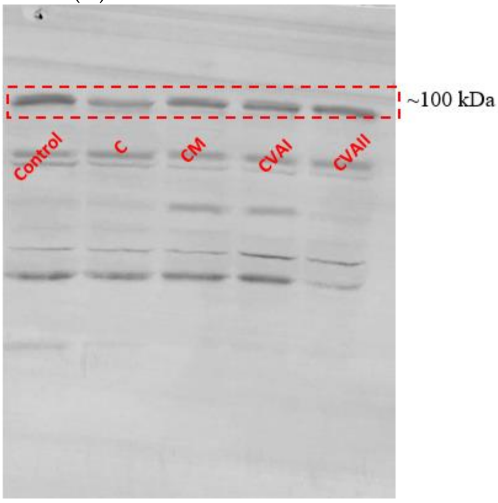

GluN2A (B)

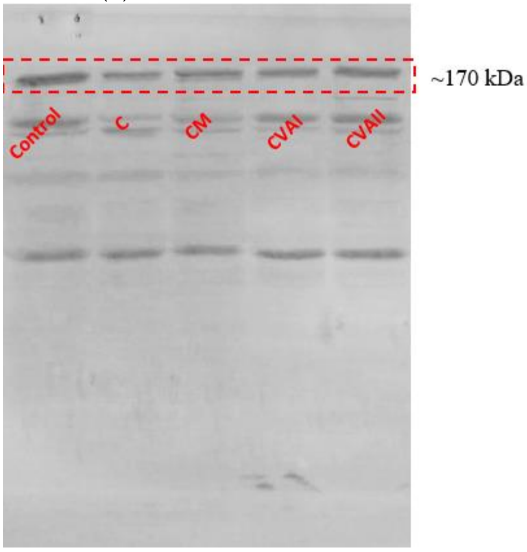

GluN1 (C)

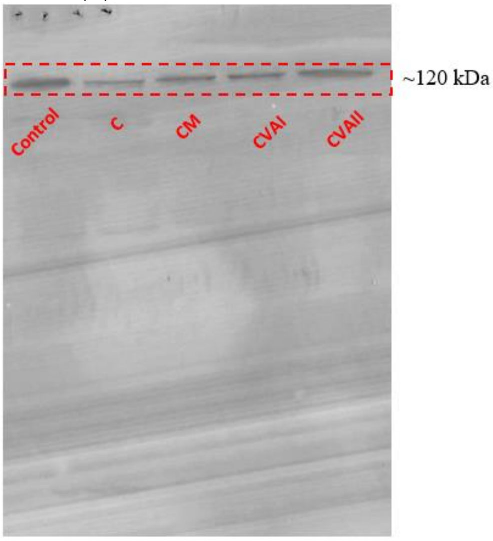

β-actin (D)

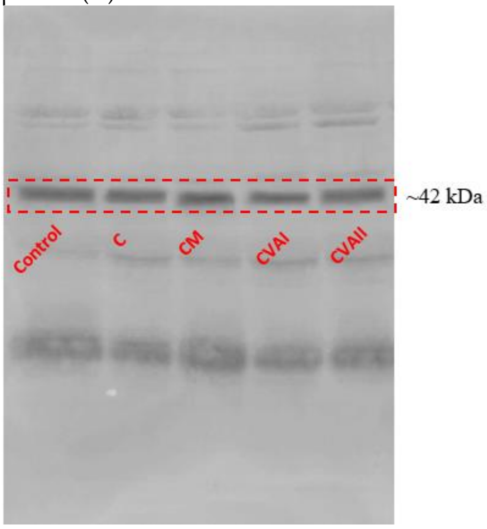

Figure S2. Full immunoblots related to Figure 5 in the main text: The effects of caffeine, melatonin, and valerian extracts (VA) on the brain tissue GluA1(A), GluN2A (B), and GluN1 (C). The densitometric analysis of the relative intensity according to the control group of the Western blotting bands was performed with β-actin normalization to ensure equal protein loading (D). Each immunoblot is a representative of three independent experiments. Red dotted rectangles delineate the results shown in Figure 5 in the main text. M.W. (in kDa) is indicated. Groups: Control: Saline followed by saline, C: Caffeine followed by saline, CM: Caffeine followed by Melatonin (2 mg/kg), CVA I: Caffeine followed by VA-Omni (100 mg/kg), and CVA II: Caffeine followed by VA-Omni (300 mg/kg).

Bax (A)

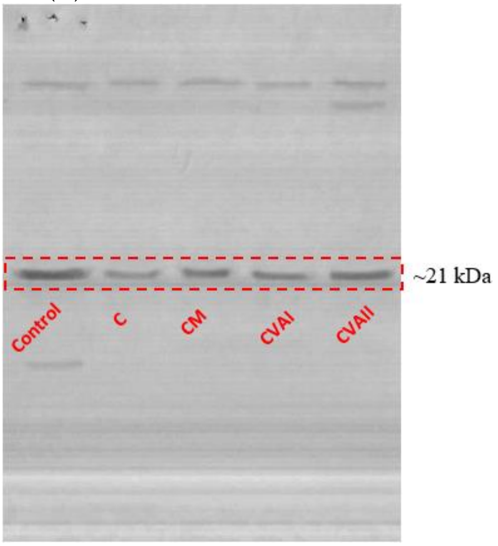

Bcl-2 (B)

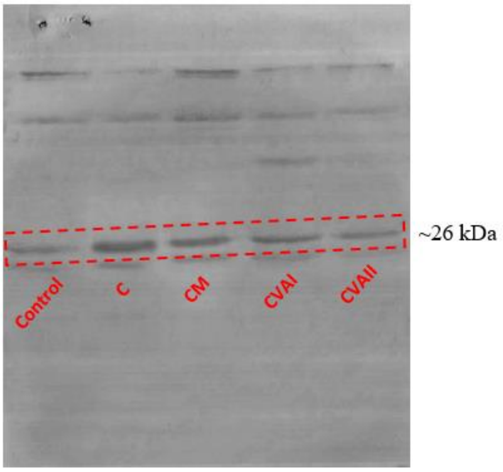

Caspase-3 (C)

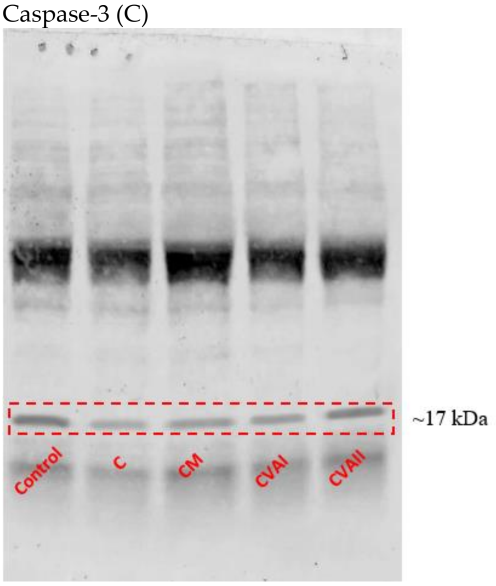

β-actin (D)

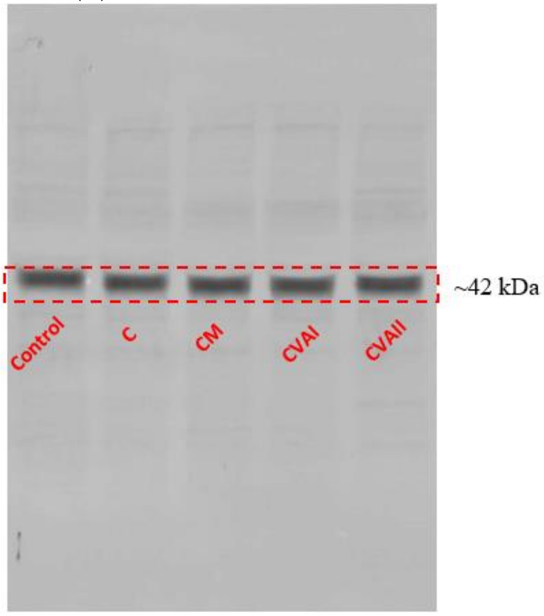

Figure S3. Full immunoblots related to Figure 6 in the main text. The effects of caffeine, melatonin, and valerian extracts (VA) on the brain tissue Bax(A), Bcl-2 (B), and Caspase-3 (C). The densitometric analysis of the relative intensity according to the control group of the Western blotting bands was performed with β-actin normalization to ensure equal protein loading (D). Each immunoblot is a representative of three independent experiments. Red dotted rectangles delineate the results shown in Figure 6 in the main text. M.W. (in kDa) is indicated. Groups: Control: Saline followed by saline, C: Caffeine followed by saline, CM: Caffeine followed by Melatonin (2 mg/kg), CVA I: Caffeine followed by VA-Omni (100 mg/kg), and CVA II: Caffeine followed by VA-Omni (300 mg/kg).
